# Mining corridors as emergence interfaces: an evidence-informed one health operational framework for prevention of infectious disease outbreaks in Kédougou, Senegal

**DOI:** 10.3389/fpubh.2026.1836395

**Published:** 2026-06-09

**Authors:** Bocar Sow, Bacary Djilocalisse Sadio, Maryam Diarra, Alioune Gaye, Marie Henriette Dior Ndione, Diawo Diallo, Serge Freddy Moukaha Doukanda, Safiétou Sankhe, Ousmane Faye, Ndongo Dia, Cheikh Talla, Oumar Faye, Cheikh Loucoubar, Gamou Fall, Boubacar Diallo, Abdourahmane Sow, Ibrahima Socé Fall, Mawlouth Diallo, Mamadou Aliou Barry, Moussa Moïse Diagne

**Affiliations:** 1 Public Health Direction, Institut Pasteur de Dakar, Dakar, Senegal; 2 Department of Public Health and Preventive Medicine, Faculty of Medicine, Pharmacy and Odonto-stomatology, Université Cheikh Anta Diop de Dakar, Dakar, Senegal; 3 Virology Department, Institut Pasteur de Dakar, Dakar, Senegal; 4 Epidemiology, Department of Clinical Research and Data Science, Institut Pasteur de Dakar, Dakar, Senegal; 5 Medical Zoology Department, Institut Pasteur de Dakar, Dakar, Senegal; 6 Chief Executive Office, Institut Pasteur de Dakar, Dakar, Senegal

**Keywords:** genomic surveillance, infectious disease emergence, Kédougou, mining corridors, one health, outbreak prevention

## Abstract

Artisanal and small-scale gold mining has rapidly reshaped southeastern Senegal's Kédougou region from a sparsely populated savanna-forest landscape into a highly connected frontier marked by land-use change, intense population mobility, and fragile basic services. Over the past decade, this corridor has recorded clustered outbreaks and warning signals involving mosquito-borne, tick-borne, and water-associated infections, including sylvatic dengue, yellow fever, chikungunya, Crimean-Congo haemorrhagic fever, and hepatitis E. These events suggest that mining settlements can function both as amplification sites, where ecological disruption, mobility, and weak services increase transmission risk, and as detection nodes, where clustered febrile illness may reveal emerging threats early. Using Kédougou as a case example, this Conceptual Analysis synthesizes outbreak experience, operational lessons, and published evidence to propose an evidence-informed One Health operational framework for mining corridors. The framework brings together fever triage and specimen referral, targeted vaccination for mobile populations, frontier-adapted mosquito and tick surveillance and control, minimum water and sanitation standards, community engagement, and joint One Health investigations supported by selective genomic sequencing when operationally useful. Rather than reporting an implemented intervention, this paper offers a structured prevention model for prospective adaptation, implementation, and evaluation in mining-affected frontier settings. The framework is intended to help districts strengthen routine services, support prevention of spillover and amplification, and improve cross-border preparedness in ecologically and socially dynamic mining corridors.

## Introduction

Mining can stimulate economic activity, but it can also create conditions that favor infectious disease emergence, rapid spread, and delayed detection. Kédougou, in southeastern Senegal, exemplifies this risk. The region lies at a savanna-forest interface with long-standing sylvatic transmission cycles for several arboviruses. It also borders Guinea and Mali, and porous crossings create a shared epidemiological space where prevention and vaccination can be uneven and difficult to sustain. As artisanal and small-scale gold mining expanded, deforestation, settlement growth, and cross-border movement intensified, bringing large numbers of workers, often young adults, into high-exposure settings with limited basic services.

These transformations are not only environmental or economic; they also reshape the conditions under which pathogens spill over, circulate, and are detected. In mining-affected frontier settings, ecological disruption, settlement informality, mobility, and weak access to diagnostics and prevention can interact in ways that amplify both infectious disease risk and the likelihood of delayed response. Kédougou is therefore a particularly instructive setting for examining how repeated outbreak signals emerge at the intersection of landscape change, population movement, and fragile routine services.

Although previous studies have documented environmental change, occupational exposure, vector ecology, and broader health risks in mining settings (1–5), the current literature remains fragmented. Much of it describes hazards, single pathogens, vector ecology, or community health impacts, while relatively little work translates these insights into an integrated, district-level operational framework for outbreak prevention and early response in mining-affected frontier settings. Existing One Health and surveillance-oriented contributions provide useful building blocks (6–10), but, to our knowledge, they do not yet provide a practical framework tailored to mining corridors as epidemiological interfaces. Our contribution is therefore not simply to describe mining-related risk, but to synthesize heterogeneous outbreak signals, contextual drivers, and operational lessons into a practical One Health framework designed for prospective adaptation, implementation, and evaluation.

In this Conceptual Analysis, we use Kédougou as a case example to synthesize outbreak signals, contextual evidence, and operational experience into an evidence-informed One Health operational framework for mining corridors. Rather than reporting a field-tested intervention, we propose a structured prevention and early-response model designed to support district-level action through routine systems, with selective use of genomics where it adds operational value. By linking outbreak signals, contextual evidence, and operational experience, the paper aims to move from recognition of risk to a more actionable framework for prevention, preparedness, and coordinated response in mining-affected frontier settings.

### Conceptual approach, case selection, and framework development

This article is a Conceptual Analysis that develops an evidence-informed operational framework for outbreak prevention in mining-affected frontier settings. It does not report primary data collection, participant recruitment, or implementation outcomes. Instead, it integrates four complementary evidence streams: (i) published outbreak investigations and surveillance-linked reports from Kédougou; (ii) literature on mining, environmental change, mobility, and infectious disease risk; (iii) operational experience from surveillance, laboratory confirmation, and outbreak response; and (iv) governance and implementation considerations relevant to district-level public health action. The aim is not to test an intervention, but to translate recurrent risk patterns into a structured framework that can support prospective adaptation, implementation, and evaluation.

Kédougou was selected as the case example because it is a particularly informative frontier setting where artisanal and small-scale gold mining, ecological disruption, cross-border mobility, long-standing sylvatic transmission cycles, and repeated outbreak signals intersect. It is also a setting where several of the authors have direct experience in surveillance, diagnostics, and outbreak response, helping anchor the analysis in both operational realities and the published literature. The outbreak examples were therefore selected not as a statistically representative sample of mining settings, but as analytically relevant events that reveal recurring transmission patterns and operational bottlenecks across mosquito-borne, tick-borne, and water-associated infections.

The framework was developed by linking recurrent risks and operational constraints identified in this evidence base to corresponding prevention and response functions. Repeated febrile clusters and diagnostic delays support standardized fever triage and specimen referral; immunity gaps in highly mobile populations support targeted vaccination; ecotonal vector exposure supports frontier-adapted mosquito and tick surveillance and control; hepatitis E outbreaks support inclusion of minimum water, sanitation and hygiene measures; and zoonotic signals support joint human-animal-environmental investigation. In this sense, the framework is both evidence-informed and operationally grounded: it is derived from observed patterns and practical lessons and proposed as a coherent model for prospective use rather than as an already tested intervention.

### A mining frontier as an emergence interface

Kédougou, in southeastern Senegal, can be understood as an emergence interface: a setting in which ecological disruption, human mobility, animal–vector–human contact, and uneven public health capacity converge in ways that increase the likelihood of pathogen spillover, amplification, delayed detection, or a combination of these processes. Located at the savanna-gallery forest interface, Kédougou has long sustained sylvatic transmission cycles for multiple arboviruses (11, 12). Kédougou's mining zones concentrate susceptible people in ecotones where human, animal, and vector contact is high while prevention and safe care pathways are weaker than in established towns. Thousands of workers move between mining camps and home communities in Senegal, Mali, and Guinea, creating dense mobility networks (13, 14). Camps are often informal, with limited safe water, sanitation and hygiene, and waste management, and with under-resourced health services. Mining activity produces standing water in pits, containers, and disturbed landscapes, and settlement expansion increases contact with wildlife at forest edges (15). In One Health terms, the corridor combines ecological change, mobility, behavior, and variable public health capacity in ways that can increase spillover risk and facilitate amplification once a pathogen is introduced (16–21). The presence of Niokolo-Koba National Park and high wildlife diversity further supports the plausibility of repeated spillovers and mixed transmission routes, reinforcing the need for integrated interventions (18, 22, 23).

### Outbreak signals from the Kédougou mining corridor

Kédougou's outbreak history is notable for diversity of transmission routes and repeated events in mining-affected districts. These signals show why mining settlements can be both amplification sites, where conditions increase transmission, and detection nodes, where clustered cases in accessible hubs trigger recognition (24–28).

Two additional operational signals deserve emphasis. First, linked fever clusters with repeated negative malaria tests in miners or camp-linked populations may indicate non-malarial outbreaks, including arboviral or viral haemorrhagic fever signals, and can justify early specimen referral and targeted risk communication. Second, livestock illness around camps, visible increases in ticks on animals supplying mining settlements, or intensified slaughtering and butchering can indicate elevated zoonotic risk and should trigger joint field investigations, targeted tick control on livestock, and safer animal-handling measures.

Sylvatic dengue (DENV-2), 2020. In late 2020 and early 2021, 59 confirmed dengue cases were detected in Kédougou (24). Cases were mainly young adult men, consistent with occupational exposure in forest-fringe mining settings where outdoor work, evening social activity, and sleeping near forest edges increase mosquito contact (29, 30). Key Aedes vectors were implicated (*Aedes furcifer, Ae. taylori, Ae. luteocephalus*) (31). Confirmation relied on molecular testing and serology, and genomic evidence supported spillover from a sylvatic dengue lineage rather than sustained urban transmission. Work-associated clusters should be treated as actionable early signals, supported by rapid specimen referral so dengue can be distinguished from malaria and other febrile illness while control and communication can still blunt transmission. The 4S syndromic sentinel surveillance network provides one mechanism to convert such clusters into alerts linked to laboratory confirmation and response (32–34).

Yellow fever, 2020–2021. During the same period, eastern Senegal reported yellow fever cases including in Kédougou (35). Yellow fever virus circulates in sylvatic cycles in the region; mobility and immunity gaps in mining areas can increase outbreak size and visibility. Entomological investigations identified bridge vectors at forest edges, with Aedes furcifer dominant and other known vectors present (*Ae. luteocephalus, Ae. taylori, Ae. africanus*), alongside peridomestic *Ae. aegypti* and *Ae. vittatus*; rapid vaccination was central to containment. Mining corridors can accumulate immunity gaps quickly because new migrants arrive continuously and routine coverage in permanent villages may not reflect coverage in camps. In the 2010s, immunity was estimated to fall to about 49% 4 years after a preventive campaign, below the level generally needed to prevent outbreaks, partly because mining expansion drew in non-immune migrants (35).

Chikungunya, recurrent activity (2015 and 2023). Kédougou has reported recurrent chikungunya activity, including a larger outbreak in 2023 with more than 200 confirmed cases (36). The recurrence and geographic concentration provided an early signal prompting investigation. Vector studies during the 2023 event suggested that forest-associated Aedes species—including *Ae. furcifer* and other sylvatic vectors—can sustain transmission in these ecotones (36). This matters operationally: interventions focused only on domestic, indoor Aedes may underperform when exposure occurs at forest edges, work sites, and peri-camp spaces.

Crimean-Congo haemorrhagic fever, 2023. In 2023, a symptomatic Crimean-Congo haemorrhagic fever case was confirmed in a miner from the Bantaco area, one of the largest mining sites in the region (37). A rapid One Health investigation found evidence of an active local zoonotic cycle, including livestock seroprevalence and virus detection in Hyalomma ticks. Signals clustered around mining villages are consistent with a pathway where livestock movement to supply camps, combined with land-use and environmental change, reshapes tick ecology and intensifies exposure at the human–animal interface (20, 27, 37). For mining corridors, this supports joint human–animal surveillance, safer animal-handling practices, and clear clinical pathways for suspected viral haemorrhagic fever linked to timely referral and confirmation (37).

Hepatitis E, 2012–2014. A large hepatitis E outbreak in Kédougou mining communities highlighted how water, sanitation and hygiene failures can drive explosive transmission. During investigations, more than 1,600 samples were tested and 64.6% were positive, with severe outcomes among pregnant women (28). Retrospective testing of jaundice specimens collected through national yellow fever surveillance confirmed the 2014 Kédougou outbreak and identified acute hepatitis E (including genotype 2b) in other regions, consistent with mining hubs acting as amplification points that can seed wider dissemination through mobility and referral networks (38).

A note on malaria. Kédougou also has high malaria burden, and malaria co-infections can anchor clinical decision-making toward “malaria until proven otherwise” (39). This anchoring can delay suspicion of arboviruses, viral haemorrhagic fevers, or enteric outbreaks and slow response. Any mining-zone fever strategy must therefore keep malaria testing central while preserving triggers that rapidly identify non-malarial signals.

### Why mining amplifies risk

#### Land-use change and mosquito and tick ecology

Forest clearing, soil disturbance, and mining pits create standing water and microhabitats suitable for mosquito breeding (40). Waste accumulation and water storage, often unavoidable in camps, can further increase breeding opportunities (41). Bridge vectors that bite wildlife and humans can thrive at camp edges, enabling spillover into concentrated human populations (12). For Crimean-Congo haemorrhagic fever, increased livestock presence and movement can raise tick density and repeatedly expose people through bites or animal contact. Reports of leptospirosis and Q fever in similar contexts reinforce the broader One Health hotspot profile (42, 43).

#### Mobility, susceptibility, and network effects

Mining attracts migrants from different areas with different immune histories and vaccination status (44). Large influxes of susceptible adults increase the chance that spillover becomes a detectable outbreak, while turnover sustains vulnerability even when nearby communities have partial immunity. Mobility simultaneously exports risk beyond the mining zone through transport corridors and return movements to home communities (45, 46).

#### Water, sanitation and hygiene (WASH) and camp infrastructure

Informal settlements commonly lack protected water sources, adequate latrines, and waste management. These deficits increase risk for enteric pathogens (47) and indirectly increase mosquito breeding through water storage and unmanaged refuse. A mining-zone prevention framework requires minimum standards and workable financing or enforcement mechanisms involving local authorities, camp leadership, and relevant private-sector actors. Even incremental improvements in water, sanitation and hygiene can substantially reduce outbreak risk (48).

#### Limited access to diagnostics and safe care pathways

Undifferentiated fever is often treated as malaria by default, while distance to reference laboratories delays confirmation for arboviral or viral haemorrhagic fever outbreaks (49). Delays allow outbreaks to grow and can lead to inappropriate clinical management and missed opportunities for safer triage and referral (50). In Senegal, however, this frontline-to-reference approach is increasingly operational through the 4S syndromic sentinel surveillance network and complementary platforms that strengthen detection and referral in high-risk districts (51–54). This provides an operational basis for embedding a mining-corridor framework within routine systems rather than building parallel structures.

### Core components of an evidence-informed One Health operational framework

An evidence-informed framework for mining corridors should combine five mutually reinforcing components.

#### Integrated fever surveillance and referral

Mining districts can adopt a standard triage approach for undifferentiated fever: test for malaria; look for outbreak signals and syndromic patterns; and trigger specimen collection and referral when criteria are met. This requires reliable specimen transport, agreed turnaround targets, and a results feedback loop so district teams can act while outbreaks are still containable. In practice, first-line diagnostics should rely on simpler and more accessible methods within a tiered system, including PCR for etiologic confirmation and serology or ELISA where appropriate. Selective genomic sequencing is proposed only for representative or epidemiologically informative confirmed samples, and only when it can answer public health questions that routine diagnostics cannot address fully, such as confirming clustering, clarifying lineage or transmission patterns, documenting importation or cross-border spread, or monitoring mutations with potential implications for diagnostics or countermeasures. In this framework, genomics is therefore intended to complement routine diagnostic approaches.

#### Targeted vaccination for mobile populations

In areas with sylvatic yellow fever risk, maintaining high vaccine coverage among miners and nearby communities is core prevention. Because mining populations change rapidly, vaccination should include periodic catch-up campaigns in mining hubs and market touchpoints, with rapid micro-campaigns triggered by surveillance signals.

#### Mosquito and tick surveillance and control adapted to frontier ecology

Programs should begin with routine, risk-based surveillance to identify dominant vectors, seasonality, and high-exposure microhabitats across mining corridors. Control should then match the exposure context through larval source management, personal protection, and focal residual spraying where appropriate. For Crimean-Congo haemorrhagic fever risk, monitoring tick abundance and animal movement can guide coordinated livestock acaricide treatment and safer animal-handling practices.

#### Minimum water, sanitation and hygiene standards as outbreak prevention

Mining settlements should aim for minimum standards for safe water access, latrine coverage, and waste disposal, supported by local authorities and private-sector engagement. Where infrastructure upgrades are slow, interim measures such as chlorination, protected storage, hygiene promotion, and rapid repair of critical water points can reduce hepatitis E risk quickly.

#### Community engagement and credible risk communication

Communication should be practical and delivered through trusted channels such as camp leaders, transport operators, community health workers, and local radio. Messages should emphasize that fever is not always malaria, early care seeking matters, vaccination protects, and small environmental actions reduce risk.

## Discussion

Using Kédougou as a case example, this Conceptual Analysis argues that mining corridors should be understood not only as areas of occupational and environmental risk, but also as epidemiological interfaces where ecological disruption, mobility, weak services, and cross-border circulation interact to create both emergence risk and opportunities for earlier detection. The repeated occurrence of mosquito-borne, tick-borne, and water-associated outbreaks in mining-affected districts supports the usefulness of a unified One Health framing rather than a pathogen-by-pathogen response model (24–28).

A central implication is that frontier mining settings do not necessarily require parallel surveillance or response systems. Instead, a feasible strategy is to adapt existing district and national mechanisms rather than create parallel systems. Key areas for strengthening include fever triage and specimen referral, targeted vaccination for mobile populations, frontier-adapted vector surveillance and control, minimum water and sanitation standards, and coordinated One Health investigation (51–54).

**Figure 1:**
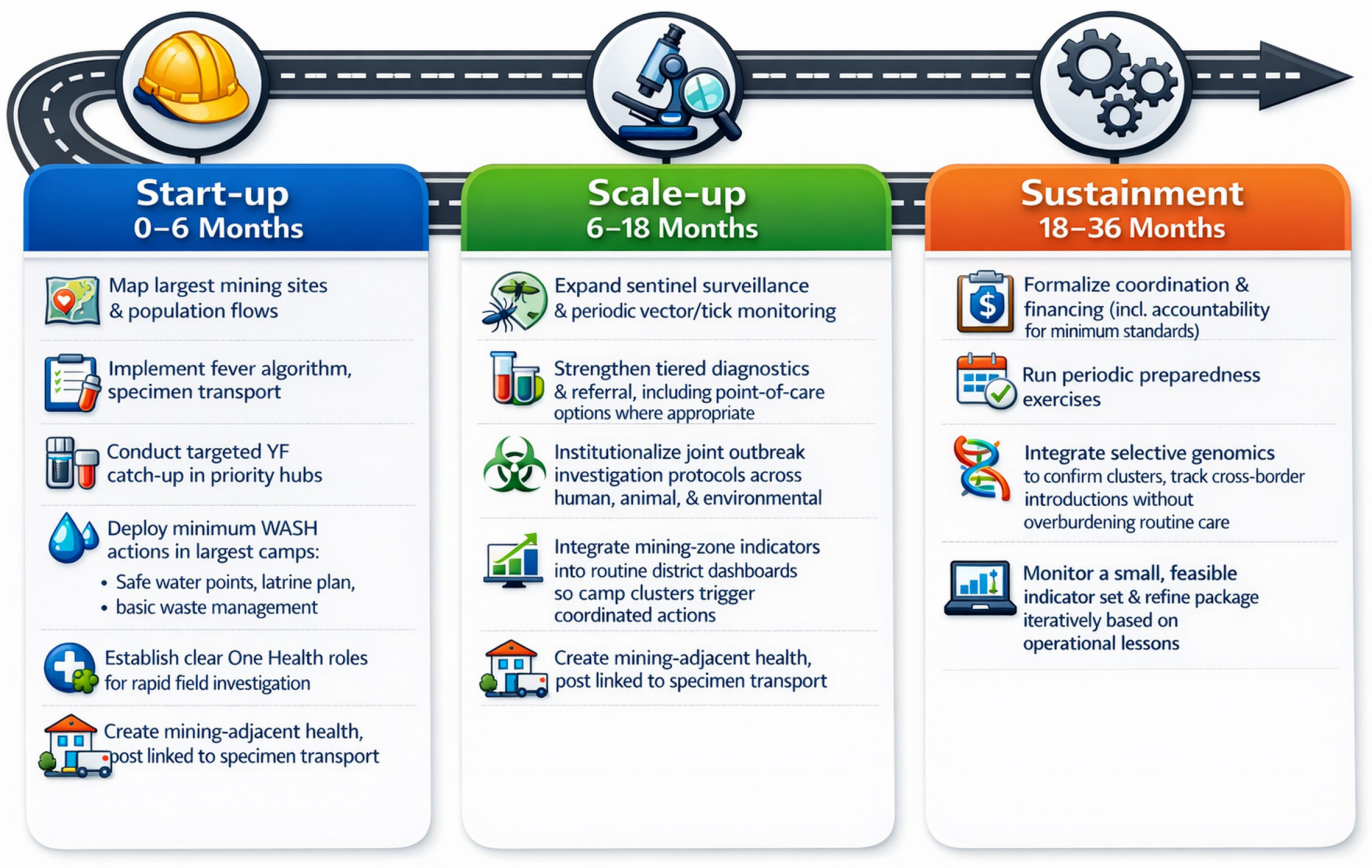
Proposed phased roadmap (0–36 months) for an evidence-informed One Health operational framework for outbreak prevention in Kédougou, Senegal. Start-up (0–6 months) includes mapping priority sites and referral routes, establishing fever triage with specimen transport, planning targeted yellow fever catch-up vaccination, and defining minimum water and sanitation actions. Scale-up (6–18 months) expands sentinel surveillance, periodic mosquito and tick monitoring, and tiered diagnostics and referral, and formalizes joint outbreak investigation across human, animal, and environmental services. Sustainment (18–36 months) aims to institutionalize coordination and financing, conduct preparedness exercises, and use selective genomics to confirm clusters and track cross-border introductions while monitoring a small indicator set.

This framework is intended to guide prospective adaptation, implementation, and evaluation rather than to claim demonstrated field effectiveness. A phased roadmap can help sequence action across start-up, scale-up, and sustainment phases (Figure 1). In practical terms, start-up should focus on mapping priority mining hubs and referral routes, standardizing fever triage, defining minimum water and sanitation actions, planning targeted vaccination in high-traffic hubs, and clarifying cross-sector roles. At this stage, district health authorities and surveillance focal points would lead mapping, referral planning, and the organization of fever triage, with support from reference laboratories and research teams for alert definitions, specimen flows, and diagnostic linkage. Local administrative authorities, community health workers, camp leaders, and, where feasible, mining-site representatives would help facilitate site access, risk communication, and implementation of minimum water, sanitation, and hygiene actions, while immunization programmes would coordinate targeted yellow fever catch-up activities in high-traffic hubs. Scale-up should expand sentinel reporting, periodic mosquito and tick monitoring, tiered diagnostics and referral, and joint outbreak investigation protocols. During this phase, district and regional health authorities, disease control programmes, veterinary and environmental services, entomology teams, and reference laboratories would lead the expansion of surveillance, vector and tick monitoring, diagnostics, and multisectoral outbreak investigation, while local authorities and decisionmakers integrate mining-related indicators into routine district dashboards and coordination mechanisms so that alerts trigger timely action. Sustainment should focus on coordination, financing, preparedness exercises, and the selective use of genomics when it changes decisions. At this stage, administrative and health authorities, policymakers, and financing bodies would need to formalize coordination, accountability, and resourcing, while laboratories and research partners contribute selective genomics, operational evaluation, and preparedness exercises. Across all phases, local communities and actors linked to mining settlements remain essential for early alerting, adherence to prevention measures, and the practical functioning of referral and response pathways. Recent governance actions also underscore the scale of informality and the need for enforceable minimum protections in mining corridors (55, 56).

The intention of this paper is not to statistically generalize from a single district, but to offer an analytically transferable framework for mining-affected frontier settings that share a recognizable set of conditions: rapid land-use change, intense mobility, settlement informality, heterogeneous immunity profiles, exposure at animal-vector-human interfaces, and delayed access to diagnostics and coordinated response. In that sense, Kédougou is used as a sentinel case rather than an exhaustive model. The framework is most likely to be relevant where these conditions co-occur, but its components would still require local adaptation depending on the dominant hazards, vector ecology, service organization, and governance environment.

Operational indicators, as used here, refer to a small set of routine, decision-oriented measures that help district systems assess whether the core functions of the framework are being implemented as intended and whether response capacity is becoming more timely and coordinated over time. Consistent with public health performance measurement and implementation literature, these indicators are intended less as exhaustive outcome measures than as practical signals for action through routine systems (57–60). Examples in this framework include timeliness and completeness of weekly sentinel reporting, specimen-to-result turnaround time, adherence to fever triage and referral, initiation of targeted yellow fever catch-up activities in high-traffic hubs, routine mosquito and tick monitoring at fixed sentinel locations, and the proportion of alerts investigated through coordinated human-animal-environmental action. Interpreted in relation to the phased roadmap outlined above (Figure 1), such indicators could support prospective evaluation while informing district-level decision-making and course correction.

During start-up, useful indicators include the proportion of priority mining hubs mapped, the existence of agreed referral and specimen transport routes, adoption of a standardized fever triage algorithm, initiation of targeted yellow fever catch-up activities in high-traffic hubs, and attainment of minimum water, sanitation and hygiene actions in the largest camps. During scale-up, indicators should focus on the performance of early warning and joint response, including timeliness and completeness of weekly sentinel reporting, specimen-to-result turnaround time, adherence to fever triage and referral, formal feedback of laboratory results to district teams, routine mosquito and tick monitoring at fixed sentinel locations, and the proportion of alerts investigated through coordinated human-animal-environmental action. During sustainment, indicators should additionally capture whether coordination and financing arrangements have been formalized, whether preparedness exercises are conducted periodically, and whether selective genomics is used when operationally justified to confirm clusters or document cross-border introductions without becoming a bottleneck. These surveillance, diagnostic, vaccination, vector, and water and sanitation indicators could be synthesized in regular district briefs to guide risk-reduction decisions and prioritize investments for miners and surrounding communities. After-action reviews could complement these metrics by documenting decision timelines, operational bottlenecks, and the extent to which cross-sector coordination occurred as intended.

Several uncertainties remain and define an important agenda for prospective implementation-oriented research. Priority questions include disentangling the relative contribution of mining-driven ecological change versus broader climate variability; producing actionable mapping of bridge-vector and tick ecology around camps; characterizing mobility networks to identify service touchpoints and cross-border vulnerabilities; assessing operational fidelity of fever triage, referral, vaccination, and WASH measures under routine district constraints; and evaluating the cost-effectiveness and feasibility of combined interventions in highly informal settings. Relevant program-based and community-based interventions could include safe water points, chlorination or household water treatment, protected water storage, latrine placement and maintenance, drainage and solid-waste management around camps, hygiene kits, handwashing promotion, and risk communication delivered through trusted local actors such as community health workers, camp leaders, and local radio. Likely targets include mining camps and peri-camp households, water collection points, food and market nodes, transport hubs, and highly mobile or otherwise vulnerable groups, because these are settings where exposure, amplification, and delayed response can converge. Although this paper does not report a completed pilot trial in Kédougou, analogous interventions in comparable hard-to-reach or outbreak settings suggest that such measures are operationally plausible and could inform future adaptation in mining-affected areas. Additional work is also needed on governance: what minimum institutional arrangements, accountability mechanisms, and financing models are required to sustain prevention efforts in mining settlements, and how district, regional, and cross-border coordination can be formalized in practice (55, 56). Selective genomic surveillance may also be valuable, but its role should remain focused on representative confirmed cases and on answering time-critical operational questions, such as confirming outbreak clustering, distinguishing circulation patterns, documenting importation, or identifying mutations relevant to diagnostics or countermeasures rather than expanding sequencing for its own sake.

PCR and serology/ELISA remain central to routine detection and confirmation within this framework, while genomics is reserved for questions where it adds clear public health value.

## Conclusion

Kédougou illustrates how rapid economic development, land-use change, ecological disturbance, and intense population mobility can reconfigure infectious disease risk at the human-animal-environment interface. The contribution of the present paper is therefore not to report an implemented intervention, but to provide an evidence-informed One Health operational framework that links outbreak signals, contextual drivers, and feasible district-level actions in a coherent prevention model. Compared with pathogen-by-pathogen approaches, this framework offers a more integrated basis for acting earlier across surveillance, vaccination, vector and tick control, WASH, and coordinated One Health investigation. Its practical relevance lies in offering districts a more coherent way to organize prevention, early warning, and coordinated response in mining-affected frontier settings, while remaining adaptable to local epidemiological and operational realities.
